# Supplementary material for: Rapid resistance development to three antistaphylococcal therapies in antibiotic-tolerant staphylococcus aureus bacteremia
Source: PLoS One. 2021 Oct 20;16(10):e0258592. doi: 10.1371/journal.pone.0258592 (PMC8528304; doi:10.1371/journal.pone.0258592)
Supplement: S1 Table — Vancomycin concentrations, dilutions tested and interpretations were performed based on the method of Sader et al. [50]. PAP/AUC ratios (test values relative to Mu3) <0.9, 0.9 to 1.3, and >1.3 are defined as VSSA, hVISA and VISA, respectively. Area under the viability-concentration curve (AUC) was determined using Microsoft Excel software and the trapezoidal method. (DOCX) [file pone.0258592.s001.docx]

**S1 Table. Population analysis profiling.** Vancomycin concentrations, dilutions tested and interpretations were performed based on the method of Sader  *et al* [50]*.* PAP/AUC ratios (test values relative to Mu3) <0.9, 0.9 to 1.3, and >1.3 are defined as VSSA, hVISA and VISA, respectively. Area under the viability-concentration curve (AUC) was determined using Microsoft Excel software and the trapezoidal method.

|  | Mu3 | BSN14S1 | BSN14S2 | BSN14R1 | BSN14R2 | BSN14RB |
| --- | --- | --- | --- | --- | --- | --- |
| AUC | 27.9±0.1 | 20.9±0.2 | 21.2±0.2 | 28.2±0.1 | 21.0±0.1 | 28.1±0.3 |
| PAP | 1 | 0.75 | 0.76 | 1.01 | 0.75 | 1.01 |
| Interpretation | hVISA | VSSA | VSSA | hVISA | VSSA | hVISA |
