# Supplementary material for: Rapid resistance development to three antistaphylococcal therapies in antibiotic-tolerant staphylococcus aureus bacteremia
Source: PLoS One. 2021 Oct 20;16(10):e0258592. doi: 10.1371/journal.pone.0258592 (PMC8528304; doi:10.1371/journal.pone.0258592)
Supplement: S2 Table — Minimum inhibitory concentrations were verified for each clinical isolate alongside identical clinical isolates from another biorepository. (DOCX) [file pone.0258592.s002.docx]

**S2 Table. Confirmatory susceptibility testing.** Minimum inhibitory concentrations were verified for each clinical isolate alongside identical clinical isolates from another biorepository.

|  | BSN14S1 | BSN14S2 | R11102 (BSN14S2) | BSN14R1 | R11153 (BSN14R1) | BSN14R2 | R11154 (BSN14R2) | BSN14RB | R11135 (BSN14RB) |
| --- | --- | --- | --- | --- | --- | --- | --- | --- | --- |
| Collection Day | 1 | 3 | 3 | 28 | 28 | 31 | 31 | 44 | 44 |
| Ceftaroline | 0.5 | 0.5 | 0.5 | 2 | 2 | 2 | 2 | 2 | 2 |
| Daptomycin | 0.5 | 0.5 | 0.5 | 4 | 4 | 0.25 | 0.25 | 2 | 2 |
| Linezolid | 2 | 2 | 2 | 1 | 1 | 1 | 1 | 1 | 1 |
| Vancomycin | 1 | 1 | 1 | 2 | 2 | 1 | 1 | 2 | 2 |
