# Supplementary material for: Rapid resistance development to three antistaphylococcal therapies in antibiotic-tolerant staphylococcus aureus bacteremia
Source: PLoS One. 2021 Oct 20;16(10):e0258592. doi: 10.1371/journal.pone.0258592 (PMC8528304; doi:10.1371/journal.pone.0258592)
Supplement: Attachment — Submitted filename: PONE-D-21-12554R1_R2R.docx [file pone.0258592.s003.docx]

Reviewer #1: The manuscript from Miller et al. documents a clinical case report of rapid resistance development to three MRSA therapies in a patient with methicillin-resistant Staphylococcus aureus bacteremia. Genetic, phenotypic and metabolic studies have been done using blood samples recovered over a six-week clinical course. The study technically sounds and this is an area of increasing interest. Authors attributed the rapid emergence of resistance to the daptomycin tolerant phenotype of the index isolate and ruled out the hypermutator phenotype through the determination of spontaneous mutation rate to rifampin in vitro. They were able to justify the appearance of mutations as a result of the antibiotic therapy that was used. It is missing from the manuscript some important information, namely: if the nostrils of the patient have been screened for staphylococcal colonization during the clinical course; the number of colonies picked at each time point and the number of colonies sequenced at each time point.
Instead of having a table in Figure 1, authors may consider to create an infographic timeline combining the information of that table with the antibiotic therapy used and including in the timeline the intermediate blood surveillance cultures negative for MRSA.

***We agree with the reviewer that important information was inadvertently omitted from the original version of the manuscript. We have indicated that nasal colonization screening was not performed at our institution (lines 67-68). We received a pure subculture from an isolated colony collected by the clinical microbiology laboratory. All colonies received from the clinical microbiology laboratory were phenotypically indistinguishable. We agree that we may have lost some genomic diversity in this step as only one isogenic colony was sequenced per day. However, we have identified a colleague who requested the same subcultures and were able to sequence those, which were identical. This is summarized in the new paragraph from lines 212-229. We have also revised Figure 1 to provide a more informative timeline.***

Minor points of criticism:
- In the abstract section, line 28 please substitute “the isolates demonstrated” by “the index isolate demonstrated”.
- Please substitute in line 108 “Patients isolates are” by “BSN14S1 is”.
- In results section, line 114 please substitute “All patient isolates” by “All comparator isolates”
- Please state the name to which the acronym BL in line 143 refers to.
- What do the asterisks in Table 2 mean?

***All proposed grammatical changes were made as requested. We thank the Reviewer for catching these errors.***

- It is missing from materials and methods the conditions of the DNA extraction for WGS.

***We apologize for this omission. DNA extraction methods have been added to the methods section***

- Did the authors used default parameters for SNP calling? If yes please state it.

***Yes, default parameters were used for the BRESEQ SNP calling. We have added a statement describing this to the methods section of the manuscript (lines 307-308).***

Reviewer #2: This manuscript reports the case of a patient with methicillin-resistant Staphylococcus aureus (MRSA) bacteremia which developed resistance to several antibiotics classically used for MRSA infections (ceftaroline and daptomycin) under the course of treatment (over a period of 44 days). Isolates also showed high minimal inhibitory concentrations (MICs) for vancomycin. Five different isolates were obtained on days 1, 3, 28, 31 and 44. These isolates were sequenced using both Nanopore MinION and Illumina. All isolates were confirmed to be isogenic. Several mutations were present in recurrent isolates compared to the initial one, in genes already described in literature as associated with resistance to daptomycin or heteroresistance to glycopeptides. Then the authors explored several hypotheses to explain the ability of this S. aureus strain to develop these mutations and to understand the potential role of the mutations detected by whole-genome sequencing (WGS). Interestingly they showed that this strain was antibiotic tolerant.


Major comments
- The results of this study are difficult to extrapolate since they only concerned one patient case but they provide insights on the potential link between antibiotic tolerance and development of antibiotic resistance. Nevertheless, data presented here did not allow to affirm that antibiotic tolerance facilitates rapid resistance development.

***We agree with the Reviewer that the data presented in the original manuscript did not affirm our argument that tolerance facilitates resistance development. We have added additional in vitro experiments to support our argument (lines 251-265, 327-334).***

- In my opinion, the manuscript is not easy to read, probably due to the fact that authors have chosen not to separate results and discussion, and it is difficult to understand what is really new in these results from that is already described in the literature. For instance, the authors described mutations in mprf or in vraT but they did not precise if the exact mutations present in this patient’s isolates have already been described and associated with antibiotic resistance or not.

***We acknowledge that the flow of the original manuscript was not ideal. We have revised the flow throughout to provide a more streamlined and easy-to-read report. Very few case reports exist that correlate tolerance and resistance development in patients with staphylococcal bacteremia and fewer still with the granularity of time points during the course of infection. We have further described how the specific mutations identified in mprF and vraT are classical mutations that have already been described and associated with antibiotic resistance.***

- Data already published about the link between antibiotic tolerance and development of antibiotic resistance should be more developed by the authors in the introduction and in the discussion. The authors should highlight in the manuscript which of their data provide new insights in the field.

***We have expanded on previous reports linking tolerance and resistance development and emphasized our data that adds to the field.***

- The authors should provide the complete antimicrobial susceptibility testing profile of the isolates (and not only oxacillin, vancomycin, ceftaroline, daptomycin, linezolid).

***We agree that the complete susceptibility profile should be included for comparison. It is added as a component of the new Figure 1.***

- They report the isolates as resistant to vancomycin but isolates have a MIC equal to 2 mg/L, which is susceptible according to CLSI recommendations. If the authors considered the isolates as heteroGISA, they should have performed population analysis profile to confirm this. So the title of the manuscript seems not adapted if the authors included vancomycin in the three antibiotics concerned by development of resistance. Moreover, authors described the appearance of a vraT mutation but the vancomycin MIC of the isolates did not increase over time so it is difficult to assign a role in antibiotic resistance to this mutation.

***We agree that per CLSI recommendations, our isolates are not VISA. We have performed PAP analysis and confirm that isolates that contain the vraT mutation are in fact hVISA. Furthermore, the specific mutation in vraT is associated with emergence of hVISA from VSSA. We have updated the text to include this information.***

- In the “Patient case” section, the authors first described the antimicrobial treatments received by the patient and then the results of bacteriological cultures. As the choice of antibiotic therapy depends on the bacteriological results, these latter should be presented before or with antibiotic therapies. Instead of figure 1 presented as a table, a chronological timeline including both bacteriological results and changes in antibiotic therapies would better illustrate the patient case.

***We agree with the Reviewer that Figure 1 as initially presented was inadequate. We have revised Figure 1 in line with the Reviewer’s excellent recommendations. We have likewise reorganized the Patient Case section throughout to provide a more understandable, sequential presentation.***

- Isolate BSN14RB showed a decreased ceftaroline MIC compared to the previous isolates: did the authors have a hypothesis about this? The authors do not discuss what would be the mechanism of resistance to ceftaroline in isolates R1 and R2.

***The antibiotic susceptibility report in the original submission contained some errors, most notably that BSN14RB remained with an elevated CPT MIC. We repeated susceptibility testing of all isolates to the four antistaphylococcal therapies used and report our findings in table S1. Furthermore, we identified isolates collected on the same day by another investigator and tested their susceptibilities which were identical. We provide additional discussion of our susceptibility testing and how it differs from the patient record in the text.***

- The method used to study the binding of cytochrome C is not described in the section “Materials and Methods”.

***We thank the Reviewer for catching this inadvertent omission. The materials and methods have been amended.***

- The authors described the selection of mutations potentially involved in the development of resistance. Did they test several colonies on the initial S. aureus population or the following isolates to check for the absence of a heterogeneous population?

***The Reviewer raises an excellent point. We received a pure subculture from an isolated colony collected by the clinical microbiology laboratory. All colonies received from the clinical microbiology laboratory were phenotypically indistinguishable. We agree that we may have lost some genomic diversity in this step as only one isogenic colony was sequenced per day. However, we have identified a colleague who requested the same subcultures and were able to sequence those, which were identical. This is summarized in the new paragraph from lines 212-229. We further discuss the potential for a heterogeneous population to rationalize the discrepancy between the susceptibilities provided in the patient record for BSN14R2 and the measured susceptibilities from the two independently-recovered isolates from that day provided to myself and my colleague.***

Minor comments
- Line 70: precise that bacteria were MRSA
- Line 121: results of doubling time are presented in Figure 2 and not Table 2
- Lines 213, 217, 244: please italicize S. aureus
- In the text, define the abbreviations TBA and MDK

***We thank the Reviewer for catching these errors. We have updated the text accordingly***.
