# Supplementary material for: Rapid resistance development to three antistaphylococcal therapies in antibiotic-tolerant staphylococcus aureus bacteremia
Source: PLoS One. 2021 Oct 20;16(10):e0258592. doi: 10.1371/journal.pone.0258592 (PMC8528304; doi:10.1371/journal.pone.0258592)
Supplement: Attachment — Submitted filename: PONE-D-21-12554R2_R2R.docx [file pone.0258592.s004.docx]

Reviewer #1: Authors have addressed reviewers' comments and they still have added additional in vitro experiments to support their arguments, namely, that antimicrobial tolerance may promote the development of antimicrobial resistance. There are still some changes authors can made in this version of the manuscript for its improvement.

*We thank the Reviewer for the additional recommendations and hope that we have adequately addressed them in this Revision.*

1. Lines 78-79 - Please clarify. Based on Figure 1 after the 11th day and for two weeks there was no weekly surveillance cultures.

*You are correct, weekly surveillance cultures were not performed as recommended in the patient notes. We have revised line 79 replacing “on weekly surveillance cultures” to read as “on subsequent cultures (days 10 and 11)”*

2. Line 84 - "Blood cultures cleared day 31", shouldn't be day 33?

*You are correct, we had mistakenly indicated day 31, the last day of culture positivity, as the first day of cleared blood culture. We have revised this line to indicate the first day of documented culture clearance as day 33.*

3. Authors refer that they perform PAP analysis but these results are not shown.

*We have added Supplemental Table 1 to explicitly provide the PAP analysis results.*

4. Please avoid referring to the isolates by the month of their isolation. Please use instead the day of the isolation or the name of the isolate to be in accordance with Figure 1.

*We have revised throughout to avoid use of months when referencing isolates.*

5. Lines 289-291 - Only volumes used were provided, no reference for the concentration of the solutions used is provided.

*Concentrations of reagents have been added*

6. Line 332 - "0.25 mg/L or 0.5 mg/L" of what?

*These were concentrations of daptomycin and the word had been removed in error. We thank the reviewer for catching this omission and the abbreviation “DAP” has been added as intended.*

7. Figure 1 - Please define antibiotics' abbreviations in the legend.

*Antibiotic abbreviations have been defined as requested.*

8. Figure 4 - In material and methods is referred that three biological replicates of each strain have been used. As so, why are represented 6 replicates (n=6) for BSN14S1?

*In order to increase the rigor of the comparisons, after isolate BSN14S1 demonstrated a more rapid emergence of DAP resistance than comparators, we performed a second, independent analysis in triplicate from a separate freezer stock of the strain. This replicated experiment was consistent with the earlier findings of more rapid emergence of DAP resistance. This demonstrates that the initial findings are reproducible.*

Reviewer #2: The authors have addressed all the points raised during the first review. Thus the clarity and robustness of the manuscript’s conclusions have been strongly improved.

*We thank the Reviewer for the kind words and hope these additional changes further strengthen the manuscript*.

I have some minor comments.
- Line 19: index isolate is not susceptible to trimethoprim/sulfamethoxazole so change “all (non-beta-lactam) antibiotics” for “almost all” or “all… except trimethoprim/sulfamethoxazole”

*We agree and have amended the abstract to include SXT resistance in the strain description.*

- Line 23: isolate BSN14RB is not susceptible to ceftaroline (table S1, MIC=2, as previous isolates R1 and R2)

*You are absolutely correct and we apologize for the oversight. Our first E-test for Ceftaroline in this isolate indicated an MIC of 1 for this isolate and we based our initial abstract on these data. Upon repeat testing in triplicate, the MIC for BSN14RB was 2, 2, 1.5 but we neglected to update the abstract. We thank the Reviewer and have corrected this error.*

- Line 93: genetic variations are not indicated in Figure 1

*We agree that revised Figure 1 no longer contains genetic data and have updated the text to only refer to Table 1.*

- Line 111: please indicate that data are presented In Figure 2. Results in the text are not consistent with those in Figure 2 (23-38 vs 23-28 for BSN14S1)

*Thank you for catching that typographical error. The correct numbers are in the text (23-38). Figure 2 has been updated to provide the correct distribution (23-38).*

- Line 216: data are shown in Table S2

*The “data not shown” has been updated to point toward supplemental table S2.*

- Line 332: precise that tubes contained daptomycin

*The text has been corrected.*

- Figure 1: what does ⴕ mean in the table?

*The superscript data has been defined as confirmation by E-test*

- Table S1: if the table is published, it would be useful to add the date of collection of each isolate as in Figure 1.

*Hospital day of collection has been added to the new Table S2 as requested.*
