# Supplementary material for: Rapid resistance development to three antistaphylococcal therapies in antibiotic-tolerant staphylococcus aureus bacteremia
Source: PLoS One. 2021 Oct 20;16(10):e0258592. doi: 10.1371/journal.pone.0258592 (PMC8528304; doi:10.1371/journal.pone.0258592)
Supplement: Attachment — Submitted filename: PONE-D-21-12554R3_R2R.docx [file pone.0258592.s005.docx]

Reviewer #1: The authors have addressed all the comments raised during the previous round of review. However, I still have some minor comments:

1. There is a typo in the beginning of the title of the manuscript. Please remove the letter "z".

*This typo has been corrected.*

2. Please clarify the susceptibility profile of the blood cultures mentioned in lines 22-23. In accordance to figure 1 and table S1, the blood culture remained with intermediate resistance to vancomycin and resistant to daptomycin and ceftaroline susceptibility was not available for this blood culture in the patient chart.

*We agree with the reviewer that it is difficult to convey susceptibility profiles to multiple antibiotics and multiple isolates in a concise narrative format. We agree that the information available to clinicians indicated probable VAN heteroresistance, DAP resistance and an unknown CPT susceptibility profile for the “RB” isolate. Ceftaroline susceptibility by Etest was ordered but the patient had been discharged before completion of the add-on testing and this test was canceled. Subsequent testing performed in table S1 confirmed the CPT intermediate resistance indicated in the text. While the ceftaroline susceptibility profile was not known to clinicians at the time, ceftaroline intermediate resistance was highly suspected due to previous cultures and confirmed by our group in table S1. Lines 23-24 have been modified to provide more precise descriptions.*

- Line 160 - Please remove the word "in".

*This typo has been corrected.*

- The interpretation criteria used to define vancomycin phenotype in table S1 (VSSA, hVISA or VISA) should be provided, as well as the PAP graphics. In table S1 why was isolate BSN14S2 considered as VSSA and isolates BSN14R1 and BSN14RB as hVISA?

*Interpretive criteria for PAP testing is a standard defined by the reference cited in the methods and in the table legend. We have reproduced the definition in the table legend. PAP graphics are not typically provided in manuscript analogous to photographs of Etest strips not typically provided in manuscripts. We have added a PAP graphic for review but do not feel it is informative for publication. Excel software was used to determine trapezoidal AUC. We note that the order of the data presented in table S1 previously was in error and thank the Reviewer for catching this oversight. The AUCs were not accurately presented to the reader. This has been corrected in the revised manuscript.*

- Please provide the name of the software used for the determination of AUC.

*We used Excel to calculate the AUC based on the formula (y_n+1_+y_n_)/2 × (x_n+1_-x_n_) where “y” is the log-transformed CFU/ml measured at consecutive concentrations of vancomycin “x”. This series of trapezoidal AUCs were then summated across the measured concentrations.*
